# Decreases in body temperature and body mass constitute pre-hibernation remodelling in the Syrian golden hamster, a facultative mammalian hibernator

**DOI:** 10.1098/rsos.160002

**Published:** 2016-04-13

**Authors:** Yuichi Chayama, Lisa Ando, Yutaka Tamura, Masayuki Miura, Yoshifumi Yamaguchi

**Affiliations:** 1 Department of Genetics, Graduate School of Pharmaceutical Sciences, The University of Tokyo, Bunkyo-ku, Tokyo 113-0033, Japan; 2 Department of Pharmacology, Faculty of Pharmacy and Pharmaceutical Sciences, Fukuyama University, Fukuyama 729-0292, Japan; 3 AMED-CREST, Japan Agency for Medical Research and Development, Chiyoda-ku, Tokyo 100-0004, Japan; 4 Precursory Research for Embryonic Science and Technology, Japan Science and Technology Agency, Chiyoda-ku, Tokyo 102-0076, Japan

**Keywords:** hibernation, body temperature control, body mass, cold acclimation, photoperiod, Syrian hamster

## Abstract

Hibernation is an adaptive strategy for surviving during periods with little or no food availability, by profoundly reducing the metabolic rate and the core body temperature (*T*_b_). Obligate hibernators (e.g. bears, ground squirrels, etc.) hibernate every winter under the strict regulation of endogenous circannual rhythms, and they are assumed to undergo adaptive remodelling in autumn, the pre-hibernation period, prior to hibernation. However, little is known about the nature of pre-hibernation remodelling. Syrian hamsters (*Mesocricetus auratus*) are facultative hibernators that can hibernate irrespective of seasons when exposed to prolonged short photoperiod and cold ambient temperature (SD-Cold) conditions. Their *T*_b_ set point reduced by the first deep torpor (DT) and then increased gradually after repeated cycles of DT and periodic arousal (PA), and finally recovered to the level observed before the prolonged SD-Cold in the post-hibernation period. We also found that, before the initiation of hibernation, the body mass of animals decreased below a threshold, indicating that hibernation in this species depends on body condition. These observations suggest that Syrian hamsters undergo pre-hibernation remodelling and that *T*_b_ and body mass can be useful physiological markers to monitor the remodelling process during the pre-hibernation period.

## 1. Introduction

Hibernation is an adaptive strategy characterized by a drastic suppression of metabolism, activity and body temperature (*T*_b_) that allows animals to survive during periods with little or no food availability; hibernation is widespread among mammals [1–3]. Two types of mammalian hibernators exist: obligate hibernators, such as black bears and ground squirrels, hibernate every winter under the regulation of a circannual clock whose molecular mechanisms are largely unknown [4–7] and facultative hibernators, such as hamsters, hibernate irrespective of endogenous circannual rhythms [8]. Mammalian hibernation is a fascinating phenomenon, with respect to basic biology as well as its possible application in medical sciences. Hibernators can endure many types of stressors including severe hypothermia, starvation, ischaemia–reperfusion injury and obesity, all of which are deleterious to non-hibernators such as mice and humans [9–11]. It has been suggested that obligate hibernators prepare for hibernation by remodelling their bodies during the pre-hibernation season in autumn [1,12,13]. Remodelling includes changes in energy deposition and lipid metabolism, which allows hibernators to store energy in the form of fat and use it as fuel during winter, and ensuring tolerance to extreme physiological challenges during hibernation [1,12–15]. However, the inductive and regulatory mechanisms of the pre-hibernation remodelling remain to be elucidated. Interestingly, Arctic ground squirrels (*Urocitellus parryii*) and alpine marmots (*Marmota marmota*)—obligate hibernators—exhibit a decrease in *T*_b_ long before beginning hibernation ([16,13], respectively), suggesting a thermoregulatory adjustment for hibernation in the early pre-hibernation period. Therefore, it is necessary to investigate the early events that occur in the pre-hibernation season to understand the complex physiological remodelling for hibernation.

**Figure 1.**
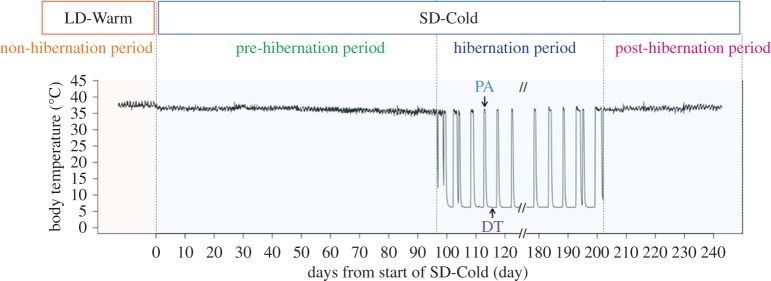
A representative example of *T*_b_ measured by *T*_b_ loggers implanted in the intraperitoneal cavity. Syrian hamsters bred in a long day photoperiod and warm ambient temperature condition (16 L : 8 D cycle, ambient temperature = 24°C, LD-Warm) were transferred to a short day photoperiod and cold ambient temperature at time 0 (8 L : 16 D cycle, ambient temperature = 4°C, SD-Cold). They began to hibernate several months after the exposure to SD-Cold, repeated DT-PA cycles for a few months and ended hibernation spontaneously while being kept in SD-Cold conditions (called the post-hibernation period). DT, deep torpor; PA, periodic arousal.

The Syrian golden hamster (*Mesocricetus auratus*) is a facultative hibernator belonging to the rodent family that can enter hibernation throughout the year when exposed to a short-day photoperiod and cold ambient temperatures (SD-Cold) for a long period (typically two to three months) under laboratory conditions [8,17,18]. Despite the differences in the physiological mechanisms regulating hibernation between obligate and facultative hibernators, the requirement of a long pre-hibernation period before the induction of hibernation in Syrian hamsters implies that these animals undergo similar pre-hibernation remodelling prior to hibernation (figure 1) [19,20]. Previous reports identified rapid tissue remodelling during the deep torpor (DT)–periodic arousal (PA) cycles in Syrian hamsters [21], but little is known about pre-hibernation remodelling in Syrian hamsters. We hypothesized that Syrian hamsters exposed to SD-Cold show pre-hibernation remodelling to prepare for the severe environmental and physiological conditions that accompany hibernation. To demonstrate that Syrian hamsters undergo pre-hibernation remodelling similar to obligate hibernators, we conducted continuous measurements of *T*_b_ with implanted *T*_b_ loggers under prolonged SD-Cold (a hibernation-inductive condition). We also examined the relationship between the pre-hibernation remodelling of the *T*_b_ set point and body mass.

## 2. Material and methods

### 2.1. Animals and housing

Three-week-old female Syrian hamsters from a closed breeding colony were purchased from SLC, Inc, Japan. Three to four animals were housed together per cage, maintained at an ambient temperature of 24–25°C with a 16 L : 8 D cycle (lights on 05.00–21.00), with ad libitum access to food (MR standard diet, Nihon Nosan, Japan) and water. Body mass of animals was measured weekly when cages were changed. One to two weeks before the transfer to SD-Cold conditions, *T*_b_ loggers (iButton®, Maxim Integrated, USA) were surgically implanted into the abdominal cavity of animals 10–15 min after pentobarbital sodium anaesthesia (65 mg kg^−1^, diluted with phosphate-buffered saline, intraperitoneal injection; Kyoritsu Seiyaku). After one to two weeks of recovery, animals were used for hibernation induction experiments. *T*_b_ loggers were recovered from animals sacrificed by decapitation 10–15 min after intraperitoneal injection of pentobarbital sodium (65 mg kg^−1^; Kyoritsu Seiyaku).

### 2.2. Hibernation induction experiments

Three experimental trials were performed to investigate changes in body mass and *T*_b_ under different conditions. Animals were reared as described above until most animals weighed over 120 g; hence, the rearing periods were different during LD-Warm conditions across the experiments (7 weeks, 13 weeks and 15 weeks for experiments 1, 2 and 3, respectively; electronic supplementary material, figures S1 and S2*a*; see below). After the animals were transferred to SD-Cold, the onset of hibernation was first detected comprehensively by the characteristic postures of animals (rolled into ball), the reduced activity and the reduced consumption of food when cages were changed. The initiation of hibernation was confirmed later by changes in *T*_b_ measured by *T*_b_ loggers in most animals. The modified ‘saw-dust method’ was also used for confirming that animals successfully hibernated [8]. Briefly, wood chips placed on the back of hibernating individuals remained in place until the animals experienced a PA. The cage replacement and body mass measurement of animals in DT were skipped to avoid disturbing DT. DT is defined as multiday heterothermy, with *T*_b_ lowered to 15°C or less [22–24].

#### 2.2.1. Experiment 1

Three-week-old female hamsters (*N* = 16) were obtained and raised as described above for seven weeks under LD-Warm conditions (16 L : 8 D cycle, ambient temperature 26°C, 15 August 2013 to 4 October 2013, until 10 weeks old). *T*_b_ loggers were implanted in 12 animals one week before transfer to SD-Cold conditions (8 L : 16 D cycle, ambient temperature 5°C). Animals were then transferred to SD-Cold conditions (4 October 2013 to 24 January 2014), and caged individually with ad libitum access to food and water. Eleven animals began to hibernate after 56–112 days (median 84 days; electronic supplementary material, figure S2*c*). The body mass of hibernating animals was between 102 and 149 g (mean 118.4 ± 3.8 g) at the start of the SD-Cold conditions (electronic supplementary material, figure S2*a*), and between 106 and 144 g (mean 121.5 ± 3.9 g) at the maximum level during the pre-hibernation period (electronic supplementary material, figure S2*b*). We sacrificed four animals that weighed over 130 g and had not hibernated between 12 and 16 weeks after the transfer to SD- Cold (obese in figure 4), to recover *T*_b_ loggers, resulting in a reduced number of animals at 16 weeks after the transfer to SD-Cold (electronic supplementary material, figure S1). The *T*_b_ loggers from three obese animals were successfully obtained. Animals that hibernated were sacrificed after repeating multiple cycles of DT and PA and *T*_b_ loggers were recovered. One animal did not hibernate and the *T*_b_ logger from that animal failed to record during the experiment.

#### 2.2.2. Experiment 2

Three-week-old female hamsters (*N* = 24) were obtained and raised as described above for 13 weeks under the LD-Warm conditions (11 March 2014 to 14 June 2014) until 16 weeks old. *T*_b_ loggers were implanted one week before the transfer to SD-Cold conditions. Animals were then transferred to SD-Cold conditions, and caged individually with ad libitum access to food and water (14 June 2014 to 4 October 2014). All the animals began to hibernate after 6–103 days (median 74 days) (electronic supplementary material, figure S2*c*). The body mass of hibernated animals at the start of the SD-Cold condition was between 112 and 171 g (mean 131.0 ± 3.4 g; electronic supplementary material, figure S2*a*), and between 101 and 147 g (mean 120.3 ± 2.7 g) at the maximum level during the pre-hibernation period (electronic supplementary material, figure S2*b*). *T*_b_ was recorded every 90 min with the logger after implantation. *T*_b_ loggers were recovered as described in Experiment 1. The *T*_b_ logger in three hibernated animals failed to record. Three animals were sacrificed after entering the post-hibernation period.

#### 2.2.3. Experiment 3

Three-week-old female hamsters (*N* = 16) were obtained and raised as described above for 15 weeks under LD-Warm conditions (10 March 2015 to 5 June 2015) until 15 weeks old. *T*_b_ loggers were implanted one week before the transfer to SD-Cold conditions. The animals were then transferred to SD-Cold conditions, and caged individually with access to food and water ad libitum (5 June 2015 to 23 October 2015). Fifteen animals began to hibernate after 49–133 days (median 77 days; electronic supplementary material, figure S2*c*). The body mass of hibernated animals at the start of the SD-Cold condition was between 120 and 168 g (mean 147.7 ± 3.9 g; electronic supplementary material, figure S2*a*), and between 110 and 156 g (mean 127.7 ± 3.7 g) at the maximum level during the pre-hibernation period (electronic supplementary material, figure S2*b*). One animal did not hibernate for the experimental term. Almost all *T*_b_ loggers failed to record.

### 2.3. *T*_b_ determination

iButtons (#DS1922 L-F5, Maxim integrated, USA) calibrated by the manufacturer were used to accurately determine the *T*_b_ to ± 0.5°C in the range of −10 to +65°C and had a resolution of 0.0625°C and a range of −40 to +85°C. All iButtons were coated with rubber (Plasti Dip, Performix^®^; total mass ∼ 3.5 g, 1.8–3.5% of animal mass) before being implanted in animals and programmed to measure *T*_b_ every 45 and 90 min in Experiments 1 and 2, respectively. In Experiment 1, *T*_b_ data were successfully obtained from four of the 11 animals that hibernated, and from three of four obese animals that had never hibernated between 12 and 16 weeks after the transfer to SD-Cold (electronic supplementary material, figure S1). To obtain more *T*_b_ data of hibernating animals, we performed Experiment 2 and obtained *T*_b_ data from 21 of 24 animals. Experiment 3 did not record *T*_b_ data for most animals possibly because of technical equipment failure.

### 2.4. Data analysis of body mass and *T*_b_

**Figure 2.**
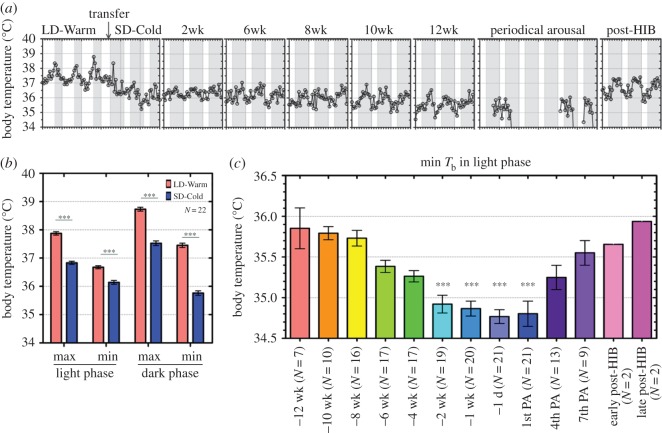
Modification of the *T*_b_ set point during the pre-hibernation period. The *T*_b_ data of 21 hibernating animals were analysed in Experiment 2. (*a*) Modification of *T*_b_ set point during the pre-hibernation period. Data from a representative animal are shown. Light and dark phases are shown as white and grey shading, respectively. (*b*) Rapid drop in *T*_b_ 1 day after the exposure to SD-Cold (8 L : 16 D cycle, ambient temperature = 4°C) conditions in both light and dark phases. Max, maximum; min, minimum. ***p < 0.005, Student's *t*-test with Welch's correction. (*c*) Retrospective analysis of the minimum *T*_b_ (min *T*_b_) during the light phase under the SD-Cold condition. –1 d and –1 wk indicate 1 day and 1 week before the first entrance into hibernation, respectively. 1st, 4th and 7th PA indicates the periodic arousal phase in the first, fourth and seventh cycles of DT-PA. Early post-HIB and late post-HIB correspond to two and four weeks after the last deep torpor (DT), respectively. *p < 0.05, **p < 0.01 and ***p < 0.005 against min *T*_b_ at –12 weeks, assessed by one-way ANOVA followed by analysis using Tukey's post hoc test. Coloured and error bars indicate mean *T*_b_ and standard errors, respectively. *N* indicates the number of animals. Post-HIB: post-hibernation period.

**Figure 3.**
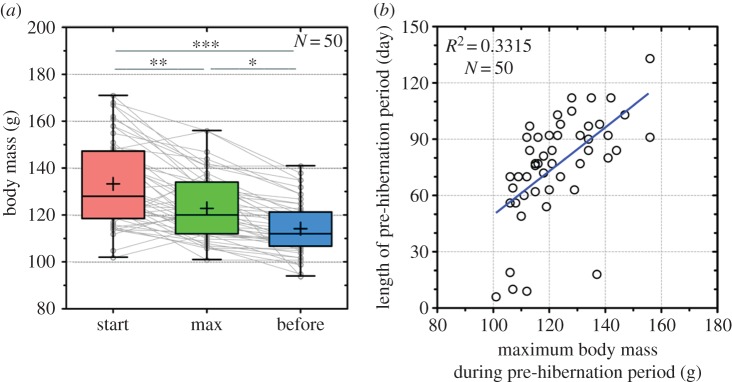
Body mass threshold for hibernation induction. Because of the lack of significant differences, we combined the data from the three experiments. (*a*) Body mass of animals at the start of exposure to SD-Cold (8 L : 16 D cycle, ambient temperature = 4°C) (start), at the maximum level during the pre-hibernation period (max) and one week before the onset of hibernation (before). Each dot represents an animal, and the lines show the changes in body mass during the pre-hibernation period. Horizontal lines and crosses indicate medians and means of body mass, respectively. Boxes enclose the interquartile ranges and whiskers show the minimum and maximum body mass at the time points. *p < 0.05, **p < 0.01 and ***p < 0.005 against body mass, assessed by one-way ANOVA followed by analysis using Tukey's post hoc test. (*b*) Positive correlation of body mass with the length of the pre-hibernation period (*R*^2^ = 0.332, *F* = 23.8 and *p* < 0.0001).

**Figure 4.**
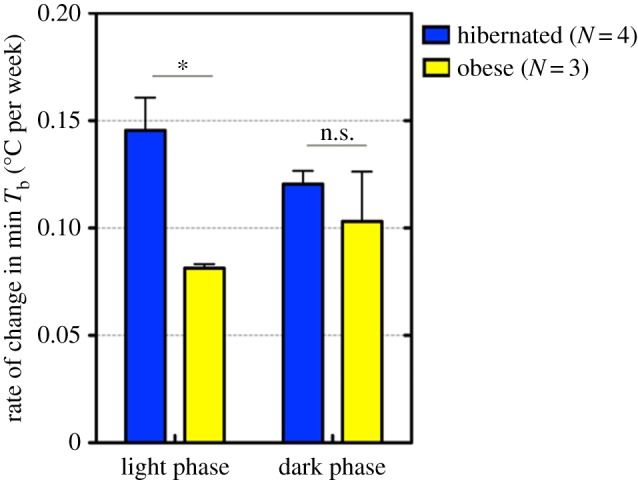
Delayed decline of the *T*_b_ set point during the pre-hibernation period in hamsters that weighed over the body mass threshold. The rate of decrease in the *T*_b_ set point during the pre-hibernation period was less in animals weighing over 130 g (obese) than those that hibernated. *p < 0.005, Student's *t*-test with Welch's correction. Error bars indicate standard errors. *N* indicates the number of animals.

The maximum body mass during the pre-hibernation period was defined as the greatest body mass during the period from one week after the transfer to SD-Cold to the first entrance into DT. No significant differences were observed in the pre-hibernation duration and the maximum body mass during pre-hibernation among the three experiments (Kruskal–Wallis test; *p* = 0.1890, and one-way analysis of variance (ANOVA); *p* = 0.247, respectively; electronic supplementary material, figure S2*b*,*c*). Therefore, we combined the data of these three experiments. The correlation of body mass with the length of the pre-hibernation period was analysed using Pearson's correlation coefficient from animals in Experiment 2 (figure 3*b*). The *T*_b_ of 21 hibernated animals in Experiment 2, including the entrance into and during hibernation, was successfully recorded and analysed. Differences in *T*_b_ were determined using a *t*-test with Welch's correction (figures 2 and 4) and a one-way ANOVA with a Tukey's post hoc test for pairwise comparisons (figures 2 and 3*a*). Statistical significance was determined at *p* < 0.05. All data were analysed using Graph Pad Prism 5.0 and Excel. Results were expressed as the means ± s.e.m. The *T*_b_ of Syrian hamsters fell to 6–7°C during the DT phase under SD-Cold conditions but recovered to a euthermic state (35–37°C) in the PA phase during the DT-PA cycle. The minimum and maximum *T*_b_ of non-, pre- and post-hibernation periods were defined as the lowest and highest *T*_b_ during the light and dark phases, respectively. *T*_b_ in PA was defined as follows: the minimum *T*_b_ (min *T*_b_) and maximum *T*_b_ (max *T*_b_) in PA were the lowest and highest *T*_b_ within 3 hours after recovery (*T*_b_ > 30°C) from the last DT and 3 hours before entry (*T*_b_ < 30°C) to the next DT, respectively. The rate of change in the min *T*_b_ for each hamster was calculated as the slope of the linear regression line of the two-dimensional scatter plot between min *T*_b_ and time (1–12 weeks in SD-Cold). The mean rate of change was compared between animals that maintained a body mass over 130 g (obese; *N* = 3) and those that hibernated (*N* = 4) in Experiment 1. In other Experiments, almost all animals gradually lost their body mass and did not maintain a body mass over 130 g after SD-Cold exposure, finally resulting in hibernation.

## 3. Results

### 3.1. *T*_b_ gradually declined after prolonged pre-hibernation

The continuous measurement of *T*_b_ from the beginning of pre-hibernation to hibernation revealed that the *T*_b_ set point of female Syrian hamsters changed acutely and chronically in response to photoperiod and ambient temperatures in Experiments 1 and 2. In Experiment 2, we succeeded in recording *T*_b_ in many hibernating animals and hence could perform statistical analysis. Transferring animals from LD-Warm into SD-Cold conditions caused an acute drop of max *T*_b_ and min *T*_b_ in both light and dark phases after 1 day (figure 2*a*,*b*). We also found that long-term exposure to SD-Cold conditions caused a chronic decrease in the *T*_b_ set point at the entrance into hibernation (figure 2*a*). To identify when such a chronic *T*_b_ decrease occurs, we performed a retrospective analysis of the *T*_b_ dataset from the timing of the first entrance into DT (figure 2*c*). This analysis demonstrated that the min *T*_b_ during the light phase decreased six weeks before the entrance into hibernation and was significantly reduced in both light and dark phases two weeks before hibernation (light phase: 34.92 ± 0.11°C, *p* < 0.005 (versus –12 weeks); figure 2*c* and electronic supplementary material, table S1). A similar response was observed for max *T*_b_ (data not shown). Reduction in min *T*_b_ and max *T*_b_ reached its lowest minimum 1 day before the first entrance into hibernation and this low *T*_b_ was maintained during PA of the first cycle of DT-PA (figure 2*c*; –1 day and 1st PA). These observations suggest that in Syrian hamsters, remodelling of the thermoregulatory system begins during the pre-hibernation period long before the onset of hibernation.

Interestingly, during repeating cycles of DT and PA in the hibernating period, *T*_b_ suppression gradually recovered (figure 2*c*; 4th PA and 7th PA), and was finally eliminated in animals that entered the post-hibernation period (figure 2*c*). Syrian hamsters kept in SD-Cold conditions spontaneously withdrew from hibernation after three to six months. This is coincident with the spontaneous recrudescence of gonadal activities during the post-hibernation period in Syrian hamsters [8] (figure 1). We observed that hamsters that recovered from hibernation exhibited higher *T*_b_ in the early (mean of two animals: 35.7°C, two weeks after the last DT-PA cycle) and late (mean of two animals: 35.9°C, four weeks after the last DT) post-hibernation periods than the late pre-hibernation period and the entrance into hibernation (figure 2*c*).

### 3.2. Body mass threshold for hibernation induction

We observed that the body mass of hamsters was crucial in determining hibernation induction. We confirmed that the body mass threshold for hibernation induction was approximately 130–140 g in independent experiments using animals obtained from breeders over consecutive years. At the beginning of SD-Cold induction, the body mass of hibernated animals (total 50 animals from Experiments 1–3) varied from 102 to 171 g (mean 133 ± 2.6 g, figure 3*a*, ‘start’). However, at a week prior to the onset of hibernation after long-term exposure to SD-Cold conditions, it decreased to between 94 and 141 g (mean 114 ± 1.6 g, figure 3*a*, ‘before’). Thus, body mass became lower than 140 g in almost all animals (figure 3*a*; electronic supplementary material, figure S3), implying a body mass threshold necessary for animals to initiate hibernation.

### 3.3. Body mass is correlated with the duration of the pre-hibernation period

Fifty animals from Experiments 1–3 required 6–133 days (median 80.5 days) in the SD-Cold condition before beginning hibernation (figure 3*b*). We found that the duration of the pre-hibernation period was significantly correlated with the maximum body mass of animals during the SD-Cold condition (*R*^2^ = 0.332, *F* = 23.8 and *p* < 0.0001; figure 3*b*; electronic supplementary material, figure S4). The ages and average of body mass of hibernated animals before transfer to the SD-Cold condition were different among Experiments 1–3 (Material and methods, electronic supplementary material, figure S2*a*). Nevertheless, the maximum body mass during pre-hibernation and the duration of pre-hibernation were similar across all experiments (ANOVA; *p* = 0.247, and Kruskal–Wallis test; *p* = 0.1890, respectively; electronic supplementary material, figure S2*b*,*c*). The loss of body mass within the first week of transferring the animals to SD-Cold condition was significantly correlated with the body mass at the start of the SD-Cold condition (electronic supplementary material, figure S2*d*,*e*; *N* = 50, *R*^2^ = 0.497, *F* = 47.5 and *p* < 0.0001). This indicated that older and larger animals lose more weight than younger and smaller animals after exposure to SD-Cold. These results suggest that age and body mass of animals before exposure to SD-Cold conditions do not affect the length of the pre-hibernation period. Several outliers (*N* = 5) in the correlation were observed in Experiment 2, as these animals hibernated within three weeks (6–19 days) of being transferred to the SD-Cold condition (electronic supplementary material, figure S2*c*, ‘Exp.2’), irrespective of their maximum body mass (figure 3*b*). However, these animals also exhibited lower body mass (94–110 g) than the body mass threshold at one week prior to hibernation (electronic supplementary material, figure S3, ‘Experiment2’). These observations suggest that body mass under SD-Cold conditions is a critical parameter for Syrian hamsters to determine whether to hibernate.

Finally, we examined the relationship between body mass and the gradual decline of the *T*_b_ set point in Syrian hamsters (figure 4). Although most animals lost body mass after prolonged SD-Cold conditions in Experiments 1–3, several animals maintained a body mass higher than 130 g even after 12 weeks of SD-Cold (obese group) and did not hibernate in Experiment 1. Interestingly, they exhibited a delay in the decline of min *T*_b_ during the pre-hibernation period; the rate of decrease in min *T*_b_ during the light phase was significantly less in animals weighing over 130 g during the pre-hibernation period than those that hibernated (*p* < 0.005, figure 4). This suggests that the body mass threshold can also affect the progression of the remodelling of the *T*_b_ set point after prolonged exposure to SD-Cold conditions.

## 4. Discussion

This study demonstrated that hibernation induction and pre-hibernation remodelling of the *T*_b_ set point in female Syrian hamsters is largely affected by body mass. The body mass threshold for hibernation induction and the correlation of the maximum body mass with the length of the pre-hibernation period suggest mechanisms controlling individual energy reserves and thereby regulating hibernation. Size of food hoards in the burrow and fat stores in the body, as well as the composition of dietary lipids, can affect the expression and duration of DT during hibernation [25–31]. In some species, large body mass tends to reduce the use of torpor during hibernation [32,33], although other studies have suggested a positive correlation between body mass and torpor expression in hedgehogs [34] and mouse lemurs [35]. The distinction of seasonal changes in body mass between species may reflect differential requirements for body fat and food hoarding during hibernation [36]. Hibernators that do not eat food during hibernation (e.g. ground squirrels, bats, hedgehogs) would require adequate amounts of energy stores in their bodies to survive in winter; therefore, they would need to increase their body mass before hibernation [37–40]. For Syrian hamsters which store and eat food during PA, a high body mass may allow the animals to endure the cold winter by consuming stored fat and cached food without going into DT and to avoid the costs associated with hibernation, thereby blocking the induction of hibernation. Conversely, a body mass lower than the threshold might force animals to enter hibernation as a beneficial strategy to reduce the consumption of the limited amounts of stored fat and food. This suggests that cost–benefit trade-offs of the use of hibernation can depend on the body mass and size of fat deposits in Syrian hamsters [41].

Syrian hamsters that hibernated were found to lose body mass and remain lean despite free access to food and water prior to the initiation of hibernation. This suggests that losing body mass after prolonged SD-Cold conditions not only is the result of an emergency response to adverse conditions, but may also reflect the progression of adaptive remodelling including the depression of energy metabolism and appetite, changes of *T*_b_ set point, and switching of energy fuels throughout the body to initiate hibernation [11,19,20,30]. Pioneering studies suggested a sliding set point hypothesis, whereby the set point of body mass gradually changed during hibernation in ground squirrels [42–44]. The hypothesis is based on the observation that the animals gain body mass at the end of summer but continuously reduce it in the hibernation season despite the presence of sufficient food in their cages. Such a sliding set point of body mass may also be present in Syrian hamsters and underlies the body mass threshold necessary for hibernation induction. Interestingly, Syrian hamsters gained body mass in response to prolonged SD in warm ambient temperatures [45]. Thus, seasonal SD promotes an increase in body mass in autumn prior to winter, and subsequent prolonged cold may induce a body mass decrease close to the sliding set point of body mass, leading to hibernation in Syrian hamsters; this is similar to the seasonal change in body mass observed in Eastern chipmunks (*Tamias striatus*) [46]. Such a system may allow the animals to determine whether to hibernate, depending on body energy reserves [47].

We found that the set point of *T*_b_ is flexible during the pre-hibernation, hibernation and post-hibernation periods in Syrian hamsters. Rapid decreases in max *T*_b_ (by 1.04 ± 0.08°C (light phase)) and min *T*_b_ (by 0.54 ± 0.08°C (light phase)) observed immediately after exposure to SD-Cold conditions may reflect a rapid adaptation to cold since the acute decline of *T*_b_ was also observed in rats exposed to cold [48,49]. Our analysis demonstrates that prolonged exposure to SD-Cold triggered a gradual decrease in *T*_b_ by two to four weeks preceding the onset of hibernation (Δmin*T*_b_ between –8 weeks and 1st PA: 0.83 ± 0.19°C (light phase)). This is consistent with a previous report that the drop in subcutaneous *T*_b_ occurred 8 days before the entrance into hibernation in Syrian hamsters [50]. Our long-term retrospective analysis further revealed that such a *T*_b_ decrease occurred long before the onset of hibernation, and *T*_b_ reached its lowest minimum (34.80 ± 0.16°C) when hibernation began in Syrian hamsters. This suggests that thermoregulation is adjusted in the early pre-hibernation remodelling period, as observed in the obligate hibernators, Arctic ground squirrel [16] and alpine marmots [13]. Interestingly, we found that Syrian hamsters gradually increased the *T*_b_ set point during repeating cycles of DT and PA. Finally, animals recovered *T*_b_ to the level of 12 weeks prior to the onset of hibernation at the post-hibernation period despite being maintained in the SD-Cold conditions. Such gradual recovery of the *T*_b_ set point during hibernation may share common physiological mechanisms with the spontaneous recovery from SD-mediated body changes that induced daily torpor after prolonged periods in Syrian hamsters [51,52]. It is noteworthy that such *T*_b_ recovery towards the end of the hibernation season is also observed in black bears [53]. However, the black bears still maintain a suppressed basal metabolic rate independently of *T*_b_ recovery, which seems different from that of Syrian hamsters that require four weeks to fully restore the suppressed *T*_b_ set point during the post-hibernation period. In future studies, it would be interesting to examine whether Syrian hamsters regulate *T*_b_ and basal metabolic rate independently.

During the pre-hibernation period, it has been suggested that physiological pre-conditioning and remodelling from a hibernation-intolerant state to a hibernation-tolerant state are induced in mammalian hibernators [1,12,13]. Our findings that lean animals decreased *T*_b_ set point more rapidly than animals that maintained a large body mass under SD-Cold conditions implies a possible effect of body mass on pre-hibernation remodelling in the *T*_b_ set point in Syrian hamsters. The physiology of the hibernation-tolerant state and its inductive mechanisms remain unclear, but they possibly involve the metabolic switch from glucose to lipid metabolism, which is preferentially activated during the hibernation period in ground squirrels and black bears [15,54]. It would also require adjustments of the central nervous system—especially the hypothalamus—that governs thermogenesis, the set point of body mass and food intake of animals [43,55,56]. Such systemic remodelling may constitute mechanisms underlying the low body mass threshold and lowered *T*_b_ set point during the pre-hibernation period. It should be noted that some animals entered hibernation much earlier than others (figure 3*b*), though the reason why remains unclear at present. One possible explanation for the existence of these animals is that they might have already finished the pre-hibernation remodelling in LD-Warm or accelerated it before SD-Cold transfer. Consistent with this assumption, previous studies showed that diet-independent remodelling of membranes precedes hibernation initiation in an obligate hibernator [13]. Moreover, lipid composition in tissues and membranes affects the propensity of torpor [27,29,30,57]. Monitoring *T*_b_ and body mass with lipid composition might be useful for identifying molecular markers and studying the mechanisms of hibernation-tolerance in Syrian hamsters, thereby leading to better understanding of the hibernation switch (to hibernate or not). In conclusion, the results of our study demonstrated that Syrian hamsters modify *T*_b_ set points during the pre-hibernation period and have a body mass threshold necessary for hibernation induction. A gradual decrease of the *T*_b_ set point may firstly facilitate survival under prolonged cold without hibernation, because it would be beneficial for animals to spare food and body fat by reducing energy demands for maintaining thermo-homeostasis in the cold. However, once the body mass and the *T*_b_ set point decrease to below threshold for hibernation induction, Syrian hamsters may start to hibernate. Thus, regulation of body mass and the *T*_b_ set point may be fundamental in the pre-hibernation remodelling in thermogenesis and energy metabolism for achieving hibernation-tolerance in Syrian hamsters. Furthermore, our observation suggests that the adjustment of the *T*_b_ set point of Syrian hamsters is reversible by the remodelling from a hibernation-intolerant to a hibernation-tolerant state, and vice versa. To determine the inductive mechanisms of remodelling at a molecular and systemic level, it is preferable to control the timing of the onset of the remodelling experimentally. This seems difficult in obligate hibernators whose hibernation is under the strict regulation of circannual rhythms, even under laboratory conditions. Using Syrian hamsters experimentally provides a unique and convenient opportunity to analyse the inductive molecular and physiological mechanisms of adaptive pre-hibernation remodelling by controlling environmental triggers (photoperiod and ambient temperature), monitoring the remodelling processes with markers including *T*_b_ and body mass, and genetic manipulation that has recently become available [58,59].

## Supplementary Material

Supplemental figures and table
