# Supplementary material for: Decreases in body temperature and body mass constitute pre-hibernation remodelling in the Syrian golden hamster, a facultative mammalian hibernator
Source: R Soc Open Sci. 2016 Apr 13;3(4):160002. doi: 10.1098/rsos.160002 (PMC4852639; doi:10.1098/rsos.160002)
Supplement: Supplemental figures and table [file rsos160002supp1.pdf]

Supplemental Fig. 1 Schematic illustration of hibernation induction experiments

Supplemental Fig. 2 Body mass difference in each experiment

(A) Body mass of animals at the start of exposure to SD-Cold (light:dark = 8:16, ambient temperature = 4°C) in each experiment (Exp.). Each dot represents an animal. Horizontal lines and error bars indicate means of body mass and standard errors, respectively. $^{**}p < 0.01$ and $^{***}p < 0.005$ against body mass, assessed by one-way ANOVA followed by analysis using Tukey's post hoc test. N indicates numbers of animals in each experiment (A-D). (B) The maximum body mass of animals during pre-hibernation period in each Exp. Each dot represents an animal. Horizontal lines and error bars indicate means of body mass and standard errors, respectively. No significant differences in the maximum body mass were observed among Exps. 1, 2, and 3 ($p = 0.247$ assessed by one-way analysis of variance (ANOVA)). (C) Box and whisker plot of pre-hibernation length in each experiment. Each dot represents an animal. Horizontal lines and crosses indicate medians and means of the length of the pre-hibernation period, respectively. Boxes enclose the inter-quartile ranges and whiskers show the minimum and maximum length of the pre-hibernation period. No significant differences in the pre-hibernation duration were noted across Exps.1, 2, and 3 ($p = 0.1890$ assessed by Kruskal-Wallis test). (D) Loss of body mass within the first week of exposure to SD-Cold condition in each experiment. Each dot represents an animal. Horizontal lines and error bars indicate means of body mass and standard errors, respectively. $^{**}p < 0.01$ and $^{***}p < 0.005$ against body mass, assessed by one-way ANOVA, followed by analysis using Tukey’s post hoc test. (E) Positive correlation of body mass loss within the first week of exposure to the SD-Cold condition with the body mass at the beginning of the SD-Cold condition indicating that larger and older animals rapidly lose more weight than the smaller and younger ones (N = 50 from Exps. 1, 2, and 3; $R^2 = 0.497$; $F = 47.5$ and $p < 0.0001$).

Supplemental Fig. 3 Body mass threshold of each experiment

Body mass of animals at the start of exposure to SD-Cold (light:dark = 8:16, ambient temperature = 4°C) ('Start'), at the maximum level during pre-hibernation period ('Max'), and 1 wk before the onset of hibernation ('Before'). Each dot represents an animal, and

the lines show the changes in body mass during the pre-hibernation period.

Supplemental Fig. 4 Correlation of body mass with pre-hibernation period of each experiment

Positive correlation of body mass with the length of pre-hibernation period (Experiment 1: N = 11, $R^2 = 0.3790$; $F = 5.493$ and $p < 0.05$; Experiment 2: N = 24, $R^2 = 0.2918$; $F = 9.063$ and $p < 0.01$; Experiment 3: N = 15, $R^2 = 0.4968$; $F = 12.83$ and $p < 0.005$).

Supplemental Table Precise data of modification of the core body temperature (Tb) set point during the pre-hibernation period in Experiment 2 (Fig.2C)

Supplemental Fig.1

Experiment1

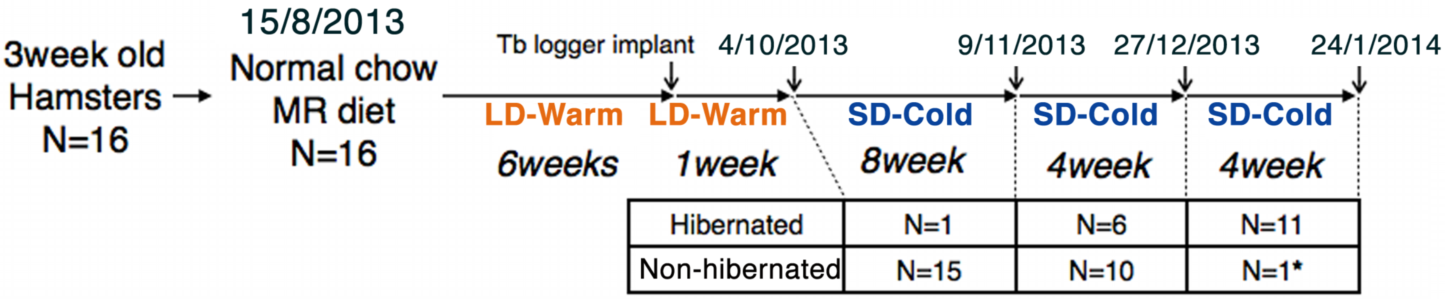

Hibernated	N=1	N=6	N=11
Non-hibernated	N=15	N=10	N=1*

Experiment2

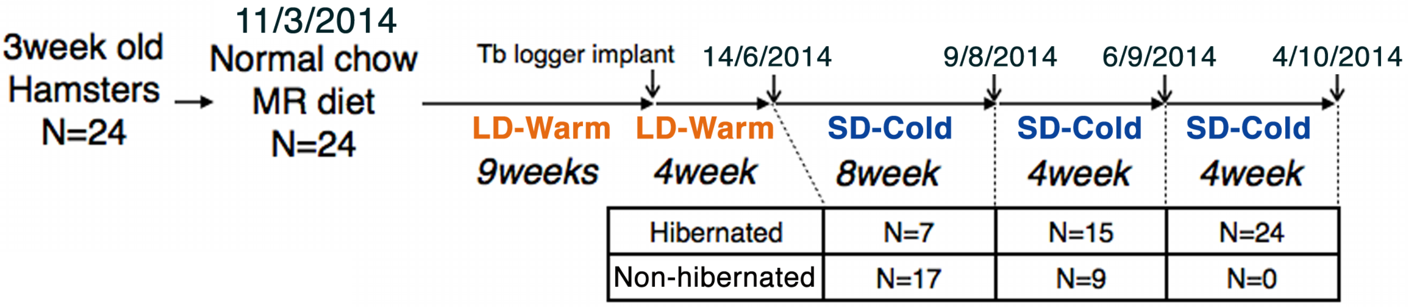

Hibernated	N=7	N=15	N=24
Non-hibernated	N=17	N=9	N=0

Experiment3

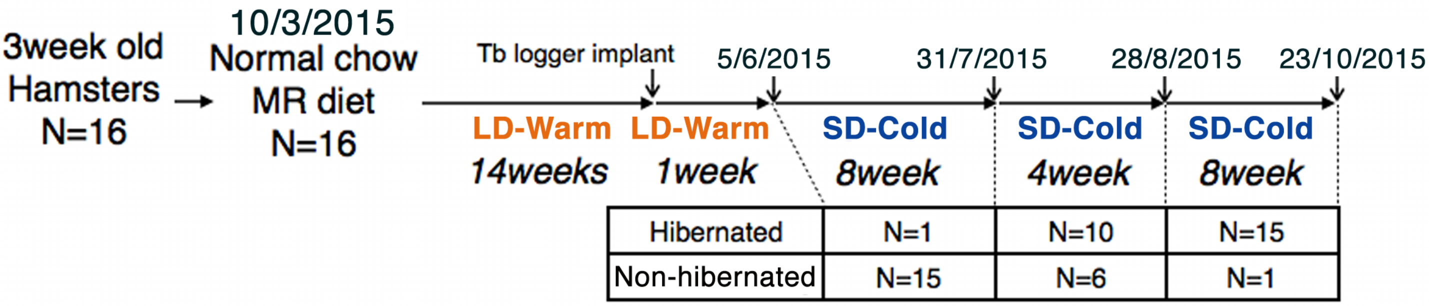

Hibernated	N=1	N=10	N=15
Non-hibernated	N=15	N=6	N=1

Supplemental Fig.2

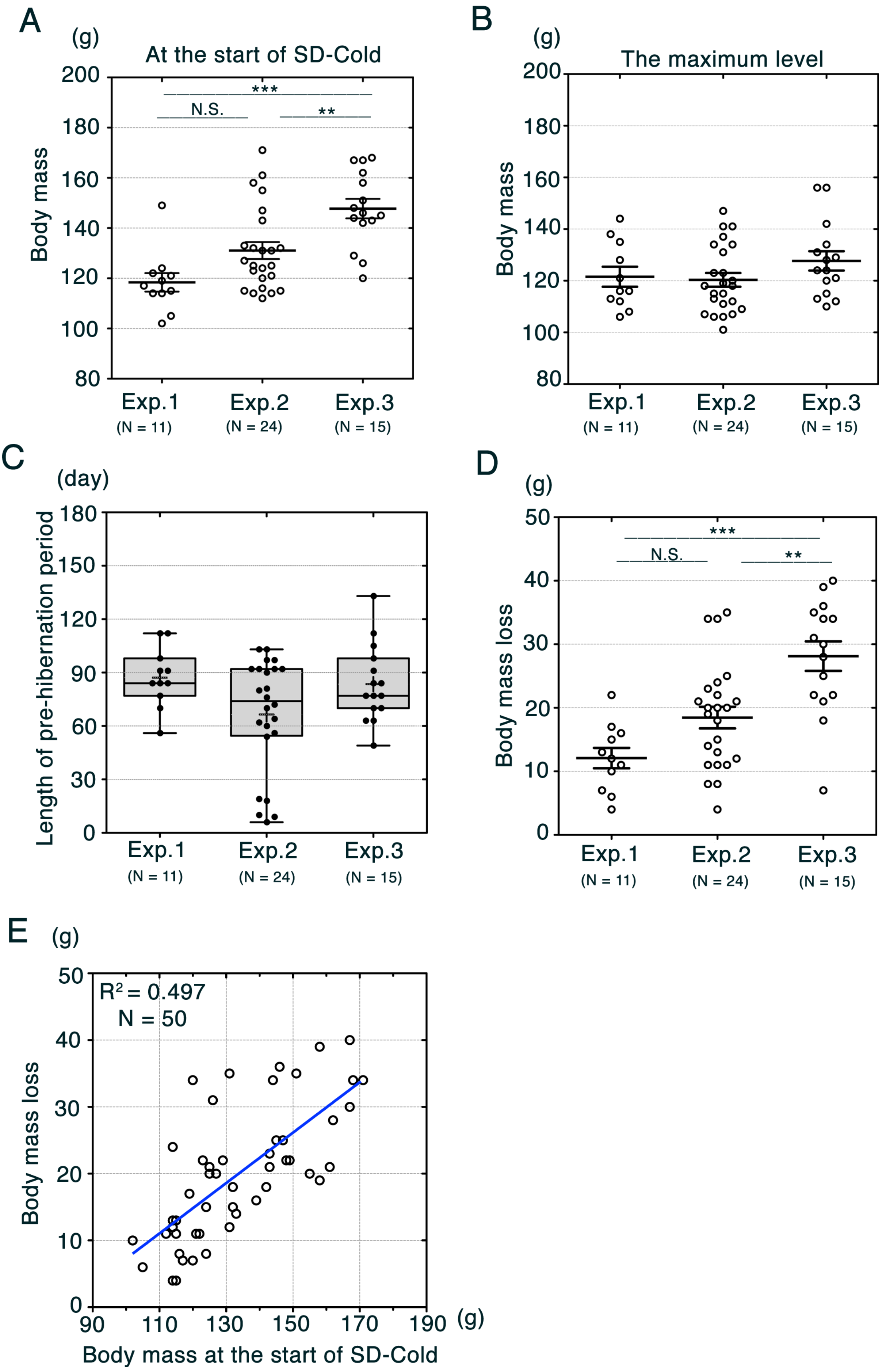

Supplemental Fig.3

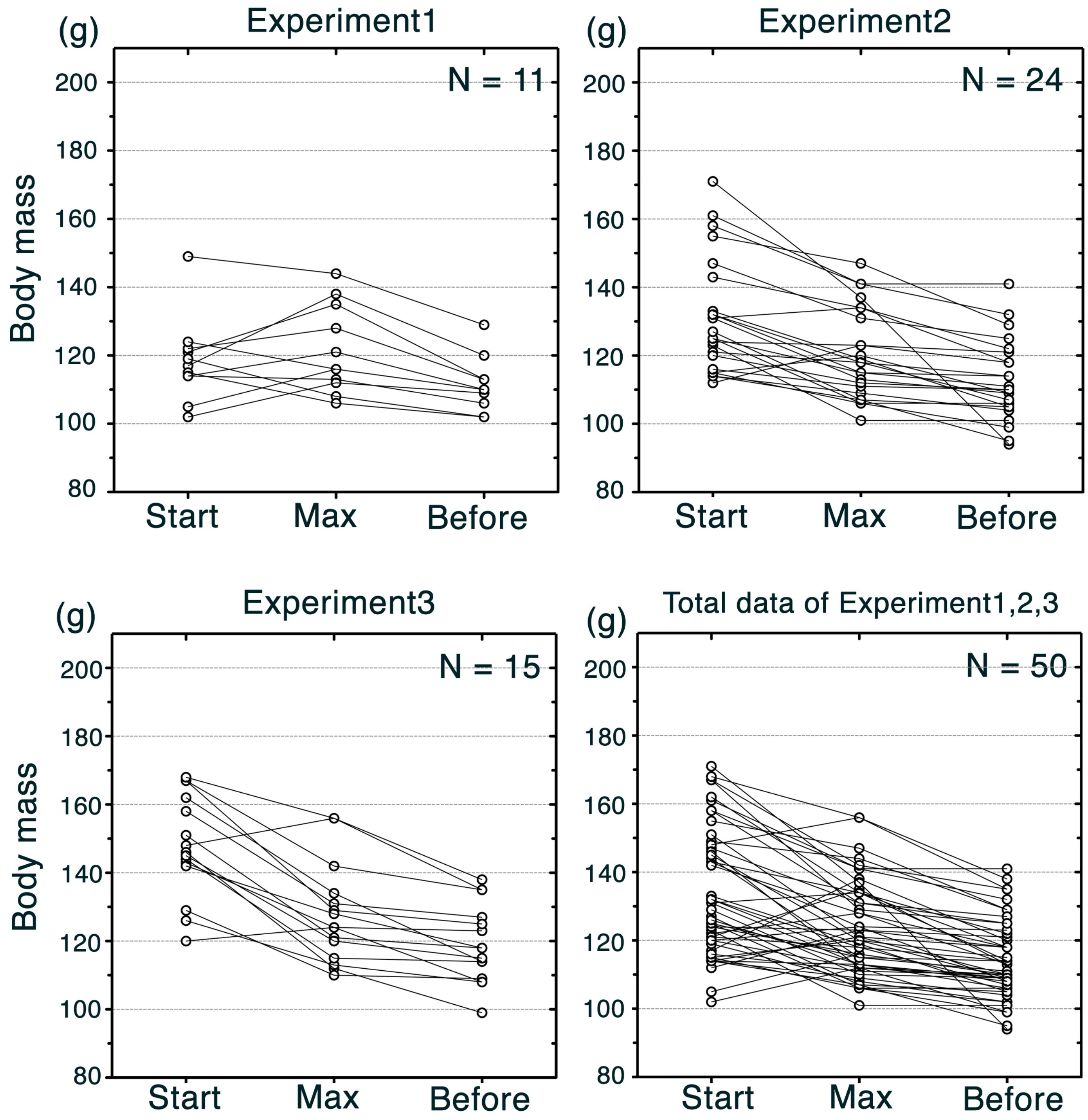

Supplemental Fig.4

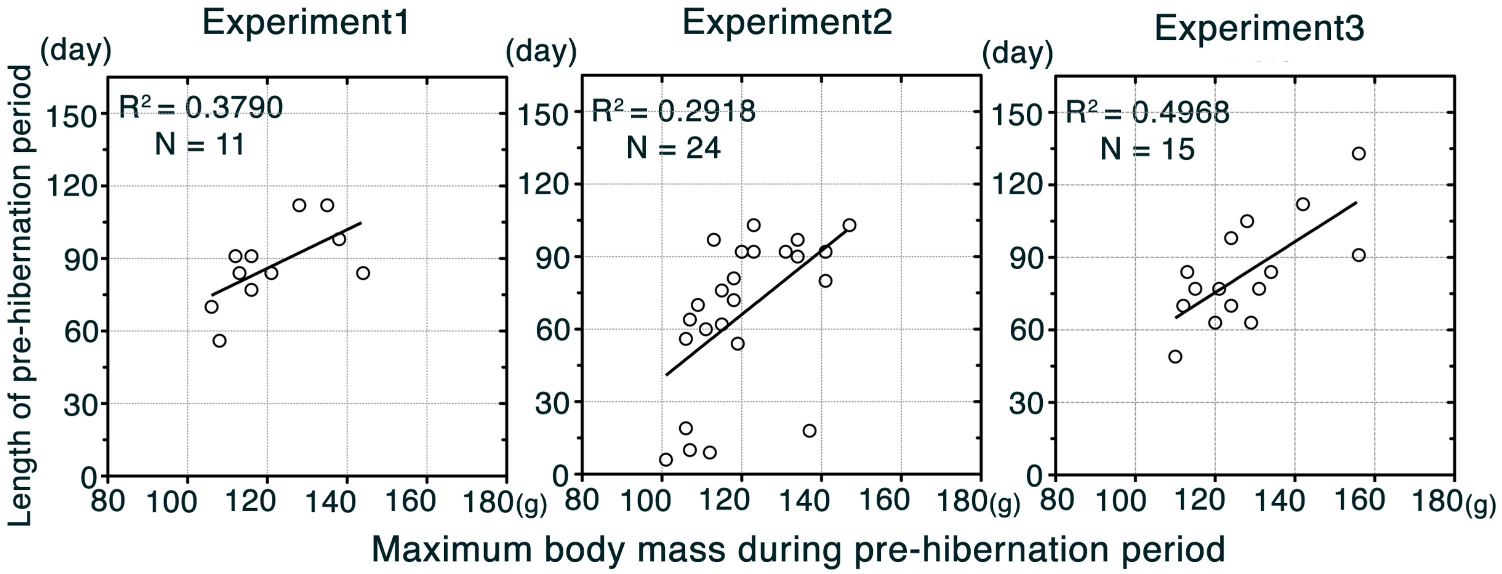

Supplemental table

Minimum body temperature at light phase (ºC)

Animal ID	-12w	-10w	-8w	-6w	-4w	-2w	-1w	-1d	1st PA	4th PA	7th PA	Early Post	Late Post	Sampling timing
Animal1	35.915	35.728	35.915	35.666	35.604	35.354	35.229	35.042	34.606		35.229	35.666	35.646	Post-HIB
Animal2	35.583	36.457	35.583	35.708	35.334	34.71	34.585	34.648	34.71	35.958	35.396	35.853	36.02	Post-HIB
Animal3			36.594	35.847	35.66	35.66	35.099	35.348	35.286					5th DT-PA
Animal4		35.022	36.082	35.646	35.396	35.209	35.459	34.835	34.398					4th DT-PA
Animal5		35.508	35.695	35.57	35.695	34.947	35.072	34.947	35.446	34.636				6th DT-PA
Animal6	36.449	36.075	35.763	35.139	34.952	35.139	34.765	34.89	34.391					4th DT-PA
Animal7	35.331	35.892	35.705	35.455	35.019	35.144	34.458	34.458	34.645					4th DT-PA
Animal8	35.363	36.172	35.923	35.799	35.736	35.674	35.674	35.301	35.114	35.736				5th DT-PA
Animal9	35.847	36.033	35.784	35.66	35.348	35.036	34.787	35.036	35.099					6th DT-PA
Animal10	35.734	35.859	35.984	35.796	35.172	34.923	35.422	34.923	34.423		35.297			12th DT-PA
Animal11			35.79	35.603	35.229	35.292	35.167	35.105	35.105	36.164				5th DT-PA
Animal12			34.574	35.509	35.322	34.823	35.26	34.137	34.885	35.197	34.948			9th DT-PA
Animal13		35.685	35.373	35.497	35.809	35.622	35.373	34.063	35.622	35.56				7th DT-PA
Animal14			35.56	35.497	35.435	35.186	35.497	35.31	34.936	34.936				10th DT-PA
Animal15			35.512	35.761	36.073	35.014	34.952	35.014	34.765	34.952	35.637			8th DT-PA
Animal16			36.059	35.872	35.622	35.435	34.936	35.31	34.749	34.811	35.622			11th DT-PA
Animal17				35.072	35.508	34.885	35.197	34.636	34.823	35.882				10th DT-PA
Animal18								34.558	35.181	34.994	35.306			13th DT-PA
Animal19						35.767	34.457	34.832	34.333					3rd DT-PA
Animal20						35.79	35.603	35.416	34.979	34.667	36.352			11th DT-PA
Animal21							35.55	35.363	35.426	34.74	36.173			12th DT-PA
Total number	7	10	16	17	17	19	20	21	21	13	9	2	2	19 (DT-PA)
Mean	35.9	35.8	35.7	35.4	35.3	34.9	34.9	34.8	34.80	35.2	35.6	35.7	35.9	7.6
SEM	0.251	0.081	0.096	0.075	0.070	0.110	0.092	0.085	0.155	0.150	0.152	-	-	0.75
